# Identification and Characterization of Erwinia Phage IT22: A New Bacteriophage-Based Biocontrol against *Erwinia amylovora*

**DOI:** 10.3390/v14112455

**Published:** 2022-11-05

**Authors:** Miloud Sabri, Kaoutar El Handi, Franco Valentini, Angelo De Stradis, El Hassan Achbani, Rachid Benkirane, Grégory Resch, Toufic Elbeaino

**Affiliations:** 1 Productions Végétales, Animales et Agro-Industrie, Faculté des Sciences, Ibn Tofail University, Kenitra 14000, Morocco; 2 Phytobacteriology and Biological Control Laboratory, Regional Center of Agricultural Research of Meknes, National Institute of Agricultural Research, Avenue Ennasr, BP 415 Rabat Principal, Rabat 10090, Morocco; 3 International Center for Advanced Mediterranean Agronomic Studies (CIHEAM of Bari), Via Ceglie 9, 70010 Valenzano, Italy; 4 Laboratory of Plant Biotechnology and Valorisation of Bio-Resources, Faculty of Sciences, Moulay Ismail University, Meknes 11201, Morocco; 5 National Research Council of Italy (CNR), Institute for Sustainable Plant Protection (IPSP), University of Bari, Via Amendola 165/A, 70126 Bari, Italy; 6 Center for Research and Innovation in Clinical Pharmaceutical Sciences (CRISP), Lausanne University Hospital (CHUV), 1011 Lausanne, Switzerland

**Keywords:** bacteriophage, *E. amylovora*, fire blight, phytopathogenic bacterium, biocontrol

## Abstract

Erwinia amylovora is a quarantine phytopathogenic bacterium that is the causal agent of fire blight, a destructive disease responsible for killing millions of fruit-bearing plants worldwide, including apple, pear, quince, and raspberry. Efficient and sustainable control strategies for this serious bacterial disease are still lacking, and traditional methods are limited to the use of antibiotics and some basic agricultural practices. This study aimed to contribute to the development of a sustainable control strategy through the identification, characterization, and application of bacteriophages (phages) able to control fire blight on pears. Phages isolated from wastewater collected in the Apulia region (southern Italy) were characterized and evaluated as antibacterial agents to treat experimental fire blight caused by E. amylovora. Transmission electron microscopy (TEM) conducted on purified phages (named EP-IT22 for Erwinia phage IT22) showed particles with icosahedral heads of ca. 90 ± 5 nm in length and long contractile tails of 100 ± 10 nm, typical of the Myoviridae family. Whole genome sequencing (WGS), assembly, and analysis of the phage DNA generated a single contig of 174.346 bp representing a complete circular genome composed of 310 open reading frames (ORFs). EP-IT22 was found to be 98.48% identical to the Straboviridae Erwinia phage Cronus (EPC) (GenBank Acc. n° NC_055743) at the nucleotide level. EP-IT22 was found to be resistant to high temperatures (up to 60 °C) and pH values between 4 and 11, and was able to accomplish a complete lytic cycle within one hour. Furthermore, the viability-qPCR and turbidity assays showed that EP-IT22 (MOI = 1) lysed 94% of E. amylovora cells in 20 h. The antibacterial activity of EP-IT22 in planta was evaluated in E. amylovora-inoculated pear plants that remained asymptomatic 40 days post inoculation, similarly to those treated with streptomycin sulphate. This is the first description of the morphological, biological, and molecular features of EP-IT22, highlighting its promising potential for biocontrol of E. amylovora against fire blight disease.

## 1. Introduction

Fire blight is caused by the bacterial pathogen *Erwinia amylovora* and is a destructive disease of pears (*Pyrus communis* L.), apples (*Malus domestica* Borkh.), and many other rosaceous plants [1]. It was first discovered in North America in the 1870s and then spread to over 50 countries [2]. Nowadays, fire blight is considered one of the most widespread and devastating diseases of plants in the *Rosaceae* subfamily *Maloideae* and a significant limiting factor for pear and apple production, since it causes huge economic losses worldwide [3]. *E. amylovora* enters the host through natural openings and wounds, colonizing xylem vessels and developing biofilm aggregates that block nutrient and water transport; it can move rapidly within the plant to establish systemic infection [4]. Most infected tissues quickly turn brown or black, as if charred by fire [5]. The pathogen affects limbs, shoots, blossoms, and, in the worst cases, the entire tree [6]. A severe outbreak can destroy an entire orchard within a single season [2].

The lack of efficient phytosanitary measures to prevent and control fire blight makes *E. amylovora* a pathogen of great concern for pear and apple producers. In the late 1990s, a failed attempt to stop the spread of fire blight on pears led to the death of 500,000 trees in Italy (Emilia-Romagna) [2]. In Romania and Croatia, a similar scenario was reported for pear, apple, and quince trees, with almost a million trees destroyed [7].

Nowadays, fire blight management mainly relies on the eradication of all infected plant tissues from affected orchards and the application of the antibiotic streptomycin [8]. However, regulatory restrictions, the emergence of resistance, and public health concerns all limit the use of antibiotics [9]. The most effective approach remains the use of trees with fire blight-resistant genotypes, but pear cultivars with high economic value are generally highly susceptible to the disease [10,11]. These limitations in controlling fire blight create an urgent need for sustainable, safe, and effective control strategies. As a treatment option, lytic phages have received great attention in recent years as potential tools for biological control of *E. amylovora*, given their systemic movement in plants and their high host specificity, unlike copper compounds and antibiotics, and the fact that they are safe to use because they are harmless to eukaryotic cells [12,13,14]. Furthermore, bacteriophages are ubiquitous and naturally occurring in the environment, self-replicating, and easily biodegradable, with some phage-based pesticides already approved for application to plants and surrounding soil or as food processing aids [15,16,17]. Therefore, phage-based biocontrol strategies can be seen as a promising and environmentally friendly alternative to streptomycin for the treatment of fire blight.

Accordingly, we report here the efficacy of the newly isolated and characterized lytic phage EP-IT22 in a planta experimental model of fire blight due to *E. amylovora*.

## 2. Materials and Methods

### 2.1. Bacterial Strains and Culture Conditions

Bacteria listed in Table 1 were grown either in liquid yeast extract peptone broth (YPG) (ThermoFisher Scientific, Monza, Italy) or on yeast extract peptone glucose agar (YPGA, i.e., YPG supplemented with 1.5% agar).

CFBP: French Collection of Phytopathogenic Bacteria, Angers, France. OMP-BO: Plant Diseases Observatory, Bologna, Italy. * Collection of CIHEAM-IAM, Bari, Italy (Valentini F., 2013; unpublished). ++: highly susceptible; +: partially susceptible; −: resistant.

### 2.2. Bacteriophage Isolation, Purification, and Titration

In December 2021, pond water and wastewater samples were collected in the Apulia region (southern Italy) (Figure S1). The collected samples were filtered through a filter paper of Grade 1, Dia. 75 × 100 mm (Whatman, Maidstone, UK) to remove large particles. Cellular debris was removed by filtration through 0.22 µm sized pores of a nylon Acrodisc^®^ syringe filter (Merck, Rome, Italy). The filtrate was centrifuged at 30,000× *g* for 1 h at 4 °C to pellet phage particles. Pellets were resuspended in 2 mL phage buffer (100 mM Tris-HCl (pH 7.6); 10 mM MgCl_2_; 100 mM NaCl; and 10 mM MgSO_4_) and mixed with 1 mL of an overnight culture of *E. amylovora* strain PGL Z1 (~10^8^ CFU/mL) in 20 mL YPG medium. After overnight incubation at 25 °C, the mixture was centrifuged at 7000× *g* for 10 min, and the supernatant was filtered through a 0.22 µm syringe filter. For initial detection of phages capable of forming plaques on *E. amylovora* in filtrates, spot tests were performed on top of a bacterial layer consisting of 200 µL of an overnight culture of *E. amylovora* strain PGL Z1 (~10^8^ CFU/mL), mixed with 6 mL of YPG soft agar (i.e., YPG supplemented with 0.7% agar), poured on top of YPGA plates. After solidification, 10 μL drops of the phage filtrate were spotted on the surface of the soft-agar layer. The drops dried at room temperature, and then the plates were incubated overnight at 25 °C. Plates were screened visually for lysis zones, and phages capable of infecting *E. amylovora* were purified from filtrates using the standard double agar overlay method [18]. Single clear plaque-forming units were transferred into 1 mL of phage buffer. This process was repeated three times to ensure the isolation of a single phage. In order to obtain high phage titers, isolated phage was amplified on the *E. amylovora* strain PGL Z1 for 24 h, poured through 0.2 µm filters, and precipitated using polyethylene glycol (PEG) 8000. Briefly, 200 mL of phage was treated with 15% (*w*/*v*) PEG 8000, gently mixed, and incubated on ice for 3 h. Bacteriophages were centrifuged at 13,000× *g* for 45 min at 4 °C, pelleted, resuspended in 5 mL of phage buffer, and stored at 4 °C. The phage titer was determined through a double-layer assay [18].

### 2.3. DNA Extraction, Whole Genome Sequencing (WGS), and Bioinformatic Analysis

Genomic DNA of isolated phage (~10^8^ PFU/mL), hereafter referred to as Erwinia phage IT22 (EP-IT22), was extracted using a DNeasy Plant Extraction kit (Qiagen, Milan, Italy). The extracted DNA was quantified using the NanoDrop™ One/OneC Microvolume UV-Vis Spectrophotometer (ThermoFisher Scientific, Waltham, MA, USA). Subsequently, 500 ng of purified genomic DNA was sent to IGA Technology services (IGATech, Udine, Italy) for Illumina sequencing (HiSeq2500 in 2 × 150 bp paired-end mode). The reads were checked for quality and trimmed using BBDUK of the BBTools package (https://sourceforge.net/projects/bbmap/ (accessed on 5 September 2022)) and assembled de novo using the Tadpole tool with different k-mers (Geneious, San Diego, CA, USA). ORFs were identified and annotated with Prokka 1.14.0 (https://Kbase.us (accessed on 11 October 2022).), and the predictions of antibiotic resistance genes and toxin-encoding genes were examined by CGE (http://www.genomicepidemiology.org/ (accessed on 21 September 2022)).

### 2.4. PCR Assays

Sense and antisense primers specific to the EP-IT22 genome were designed to investigate chromosomal structure, and sequence variation was identified through genomic comparison (Table S1). PCRs were performed with 50 ng of purified phage DNA, mixed with 2.5 µL of Dream Taq buffer 10× (Fermentas, Vilnius, Lithuania), 0.5 µL of dNTPs (10 mM), 0.5 µL of each sense and antisense primers (10 µM), and 0.25 µL of Dream Taq enzyme (5 U/µL) in a final volume 25 µL. PCR amplification was run in a Bio-Rad C1000 thermal cycler with an initial denaturation at 94 °C for 4 min, followed by 40 cycles at 94 °C for 30 s, annealing at 55 °C for 40 s, elongation at 72 °C for 40 s, and a final elongation cycle at 72 °C for 7 min. Amplified products (10 μL) were run on 1.2% TAE agarose gels, visualized on a UV trans-illuminator, and Sanger-sequenced from both directions (Eurofins Genomics, Ebersberg, Germany).

### 2.5. Host Range Determination

The spot test and turbidity assays were used to determine the host specificity of EP-IT22 on seven different strains of *E. amylovora* and three different plant pathogenic bacterial species (Table 1). For the spot test assay, 200 µL of an overnight culture of a bacterial strain (~10^8^ CFU/mL) was mixed with 6 mL of YPG soft agar (i.e., YPG supplemented with 0.7% agar), poured into YPGA plates, and maintained at room temperature for 15 min. Subsequently, a 10 μL drop of phage stock solution (~10^8^ PFU/mL) was spotted onto the surface of the plates, followed by overnight incubation at 25 °C. After incubation, the plates were visually examined for clearance (lytic) zones, whose presence was considered evidence of bacterial susceptibility to phage-mediated lysis. Lytic activity was evaluated as clear plaque (++), turbid plaque (+), and no plaque (−). For the turbidity assay, 3 mL of YPG broth was inoculated with 100 μL of an overnight culture of *E. amylovora* strains (~10^8^ CFU/mL) and 100 μL of EP-IT22 suspension at an MOI = 1. The mixture was incubated at 25 °C. One optical density measure at 600 nm (OD600) was taken after 20 h, using the NanoDrop™ One/OneC Microvolume UV-Vis Spectrophotometer (ThermoFisher Scientific, Waltham, MA, USA).

### 2.6. Microscopy

#### 2.6.1. Transmission Electron Microscopy (TEM)

To investigate phage growth kinetics, an *E. amylovora* PGL Z1 culture was challenged with EP-IT22 (multiplicity of infection, MOI = 1) at room temperature. Samples were taken at 15, 30, 60, and 120 min post-infection (pi) and observed via TEM (FEI MORGAGNI 282D, USA) using the dip method, i.e., the carbon-coated copper/rhodium grids were incubated either with phage-treated or non-treated (control) bacterial suspension for 2 min and then rinsed with 200 μL of distilled water. Negative staining was obtained by floating the grids in 200 μL of 0.5 % *w*/*v* UA-Zero EM stain (Agar-Scientific Ltd., Stansted, UK) solution and observed using an accelerating voltage of 80 kV.

#### 2.6.2. Fluorescence Microscopy (FM)

An aliquot of an *E. amylovora* PGL Z1 overnight culture was incubated with EP-IT22 (MOI = 1) at room temperature. The LIVE/DEAD^®^ BacLight™ viability kit (ThermoFisher Scientific, Milan, Italy) was used according to the manufacturer’s recommendations to assess the viability of bacteria cells treated with phages at 1, 2, and 8 h post-infection [19]. Briefly, 9 μL of samples was mixed with 0.5 μL of SYTO9 and 0.5 μL of propidium iodide (PI) for 15 min at room temperature in the dark. Photomicrographs were taken on a Nikon E800 microscope using fluorescein isothiocyanate (480/30 excitation filter, DM505 dichroic mirror, 535/40 emission filter) and tetramethyl rhodamine isocyanate (546/10 excitation filter, DM575 dichroic mirror, 590 emission filter) fluorescence filter sets.

### 2.7. Turbidity Assay

For the turbidity assay, 3 mL of YPG broth was inoculated with 100 μL of an overnight culture of *E. amylovora* strain PGL Z1 (~10^8^ CFU/mL) and 100 μL of EP-IT22 suspension at an MOI = 1. The mixture was incubated at 25 °C. Six optical density measures (0 min, 2 h, 4 h, 6 h, 18 h, and 20 h) at 600 nm (OD600) were taken in 20 h, using the NanoDrop™ One/OneC Microvolume UV-Vis Spectrophotometer (ThermoFisher Scientific, Waltham, MA, USA).

### 2.8. Viable-Quantitative PCR (v-qPCR)

As for the turbidity assays, 3 mL of YPG broth was inoculated with 100 μL of an overnight culture of *E. amylovora* strain PGL Z1 (~10^8^ CFU/mL) and 100 μL of EP-IT22 suspension at an MOI = 1. After 20 h of incubation at 25 °C, 200 µL of the mixture was transferred to a new tube and treated with PMAxx (Biotium, Rome, Italy) at a final concentration of 7.5 μM. Subsequently, samples were incubated in the dark at room temperature for 8 min, followed by photoactivation for 15 min. Genomic DNA of all samples was extracted using the cetyltrimethylammonium bromide (CTAB) method [20]. Briefly, 1 g of plant tissue was grinded with 2 mL of CTAB buffer and heated at 65 °C for 30 min. Plant residues were centrifuged at 13,000× *g* for 15 min, and the supernatant was twice washed with chloroform, and precipitated in cold iso-propyl alcohol. V-qPCR was carried out in a thermocycler apparatus (Bio-Rad CFX96, BioRad, Milan, Italy), using Ea-lsc primers and conditions reported in Laforest et al. [21]. The v-qPCR cycles consisted of an initial denaturation step at 95 °C for 5 min; 45 cycles of 95 °C for 30 s and 60 °C for 30 s with fluorescence readings at each cycle.

### 2.9. Resistance to Environmental Stressors

The stability of EP-IT22 under different pH and temperature conditions was explored. In order to assess temperature stability, phage suspensions (~10^8^ PFU/mL) were incubated separately at -80, -20, 4, 25, 40, 50, 60, and 70 °C for 60 min and phage activity was determined using the spot test assay. In the pH stability test, 100 μL of phage suspension was mixed with an equal volume of phage buffer adjusted to pH values ranging from 4 to 12 and incubated at room temperature for 1 h. Subsequently, treated phage suspensions were incubated overnight at 25 °C with *E. amylovora* PGL Z1 (~10^8^ CFU/mL) in YPG broth. The pH stability of treated phage particles was determined based on their lytic activity against *E. amylovora* PGL Z1 by measuring the OD600 after 20 h of incubation. Data were presented as mean values ± standard deviation (SD).

### 2.10. Efficacy of EP-IT22 against E. amylovora on Pear Plants

EP-IT22 in plant antibacterial activity against *E. amylovora* infection was investigated on one-year-old pear plants (*cv*. ‘Conference’) in a quarantine greenhouse under controlled conditions (25 °C and ~90% humidity). The biocontrol experiment consisted of injecting (using a sterile syringe with needle) 100 μL of an *E. amylovora* PGL Z1 suspension (~10^8^ CFU/mL) into the stem of pear plants (*n* = 10), followed 30 min later by an injection of 100 μL of phage solution (~10^8^ PFU/mL, MOI = 1) at the same site on the stem. Non-treated plants inoculated with water instead of bacteria and phages or with *E. amylovora* PGL Z1 only were included as negative and positive controls, respectively. Streptomycin sulphate-treated plants (100 μg/mL) were used as treated controls. All plants were inspected daily for 40 days post-treatment to monitor symptom development. To investigate the possible translocation of *E. amylovora* inside the stems of treated and untreated pear plants, pieces from leaves located directly over the injection sites were periodically (every 10 days) collected and tested by qPCR, according to Laforest et al. [21]. Furthermore, at the end of the experiment, stems from injection sites were collected, and the *E. amylovora* load was determined by qPCR.

## 3. Results and Discussion

### 3.1. Phage Isolation and Host Range Analysis

One bacteriophage (hereafter referred to as EP-IT22) was isolated from the collected pond water and wastewater samples, and the spot test showed that EP-IT22 exhibits high lytic activity against *E. amylovora* PGL Z1. Furthermore, the host range analysis showed that EP-IT22 was able to kill all tested strains of *E. amylovora* (Figure S2). Its inability to lyse strains of other plant pathogens (Table 1) indicated that EP-IT22 is likely specific to *E. amylovora*.

### 3.2. Biological Analysis of EP-IT22

**Figure 1:**
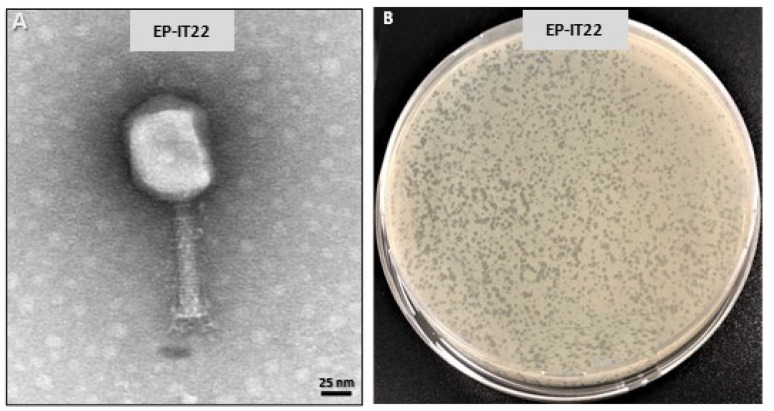
(**A**) Transmission electron microscopy (TEM) of EP-IT22 showing a particle with an icosahedral head and a long contractile tail, scale bar = 25 nm. (**B**) Double-layer assay showing EP-IT22 plaques forming units of ca. 2 mm in diameter.

EP-IT22 imaging under TEM revealed an icosahedral head ca. 90 ± 5 nm long and 75 ± 3 nm in diameter, and a long contractile tail, 100 ± 10 long and 15± 5 nm wide, with a basal tuft (Figure 1A). Based on these morphological characteristics, phage EP-IT22 was assigned to the *Myoviridae* family according to Ackermann’s classification system [22], whereas *Myoviridae* phages typically possess double-stranded (ds) DNA as their genomic nucleic acid. In addition, EP-IT22 formed clear plaques approximately 2 mm in diameter in the double-layer assay (Figure 1B), which is a characteristic of lytic bacteriophages, whereas lysogenic bacteriophages produce turbid plaques [23].

### 3.3. EP-IT22 Genomic Analysis

WGS of purified EP-IT22 genomic DNA yielded a total of 456,091 high quality reads of 150 bp. De novo assembly yielded 1094 contigs >=1000 nts long. BLASTX search of the NCBI virus database showed that our contigs are closely related to the Erwinia phage Cronus (EPC) reported in GenBank (acc. n°: NC_055743). Further analysis by mapping the Illumina reads against the EPC reference genome allowed identification of a total of 443,457 reads, mapping the full genome sequence of EPC; after complete genome sequence construction, EP-IT22 was found to have a DNA genome of 174,346 bp (compared to 175,774 bp of the EPC reference isolate). The genome sequence consisted of a G + C content of 38.4%, which is significantly lower than that of *E. amylovora* (average 53.6%). In general, the lower GC content in bacteriophage genomes is considered an adaptive strategy to optimize gene expression of the viral genome [24]. At the nucleotide level, the genome of EP-IT22 is significantly similar (*ca*. 98.48%) to that of EPC reported in GenBank. Sequencing of PCR fragments generated from EP-IT22-1 primers (Table S1), showed that the EP-IT22 genome encompasses an additional 135 bp stretch at its 5′ end that was lacking in the reference genome. This result confirmed the WGS-generated sequences from that region. In addition, the primers EP-IT22-2 designed at the beginning and end of the EP-IT22 genome yielded a PCR amplicon of the expected size (734 bp) with a 100% sequence match to the genomic sequence, demonstrating that the EP-IT22 genome is circular. Furthermore, sequences of four additional PCR amplicons (obtained with primer pairs EP-IT22-3 to -6 designed to amplify regions of genomic discrepancies between EP-IT22 and EPC (Supplementary Materials Table S1) confirmed the genomic differences found between the two phages. The genome sequence of EP-IT22 is deposited in GenBank under accession number OP586623.

**Figure 2:**
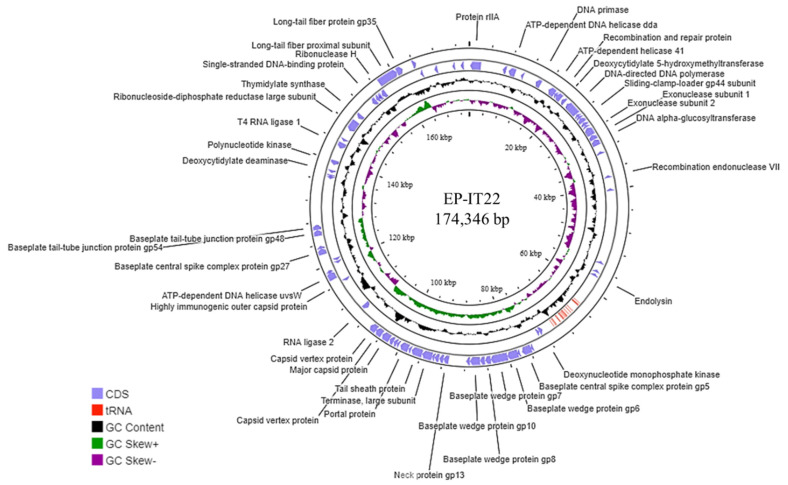
Genome map of the EP-IT22 phage, constructed with ProKsee (https://proksee.ca/ (accessed on 26 September 2022)), showing important functional proteins predicted from bioinformatic analysis. Hypothetical proteins are not reported.

The EP-IT22 genome harbored 310 ORFs, 98 of which (31.61%) had a known function, whereas the remaining 212 (68.39%) were of unknown function (Figure 2). Genome analysis showed that EP-IT22 is strictly lytic, given that it does not carry genes encoding integrases or other proteins, i.e., C1 repressor-like proteins, which are normally associated with lysogeny. CGE analysis showed that the EP-IT22 genome does not harbor any known virulence, antibiotic resistance, or toxin-encoding genes, indicating its suitability for use as a biocontrol agent.

### 3.4. Resistance to Environmental Stresses

**Figure 3:**
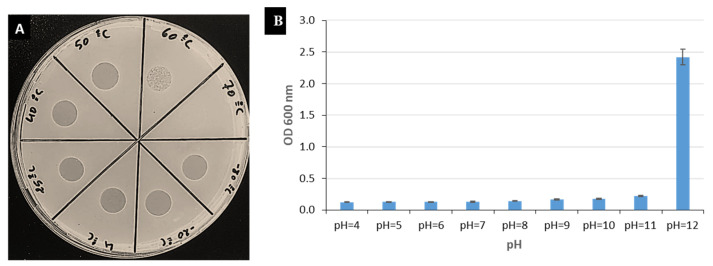
(**A**) Spot tests showing inhibition zones induced by phage EP-IT22 challenged at different temperatures (−80 °C to 70 °C). (**B**) Bacteriolytic effect of EP-IT22 treated at different pH levels (4–12) on *E. amylovora* PGL Z1 growth. Error bars represent standard deviations of three replicates.

EP-IT22 treated at different temperatures (−80 °C to 70 °C) was biologically active at temperatures ranging from −80 °C to 60 °C, whereas incubation at 70 °C for 1 h killed it (Figure 3A). Furthermore, EP-IT22 was active at pH values ranging from 4 to 11, but was inactive at pH 12 (Figure 3B). GC Skeq.

### 3.5. Growth Kinetics of EP-IT22

**Figure 4:**
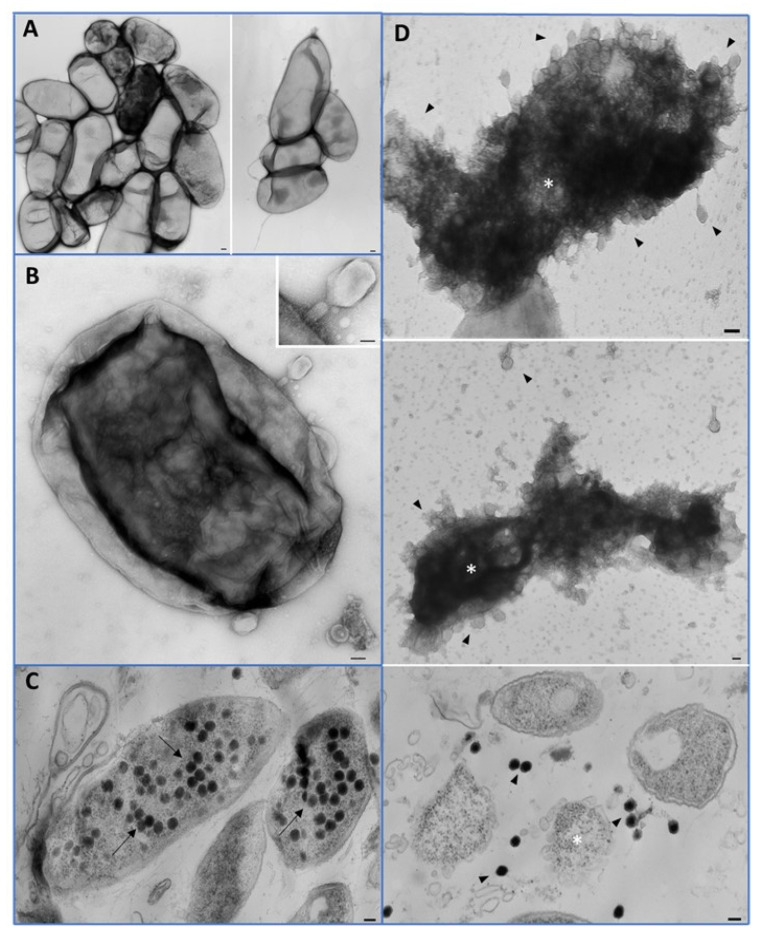
Transmission electron microscopy studies of *E. amylovora* PGL Z1 and EP-IT22 interaction. (**A**) *E. amylovora* cells, bar = 100 nm. (**B**) EP-IT22 phage adsorption on *E. amylovora* cell surface, bar = 50 nm, inset 25 nm. (**C**) *E. amylovora* cells ultrastructure with proliferation of phages (arrow), bar = 100 nm. (**D**) Complete lysis of bacteria cells (asterisk) with release of new phages (arrowhead), bar = 100 nm.

TEM was used to determine the growth kinetics of EP-IT22 through the interaction between the phage and *E. amylovora* PGL Z1 cells (Figure 4A). Phage attachment to *E. amylovora* PGL Z1 cell surfaces was confirmed visually 15 min after contact (Figure 4B), while phage progeny was already visible in the bacterial cytoplasm 30 min post-contact (Figure 4C). Lysed *E. amylovora* PGL Z1 cells and free phages surrounding lysed bacteria were visualized 60 min post-contact (Figure 4D). These observations confirmed the capacity of EP-IT22 to efficiently adsorb, infect, and replicate on *E. amylovora* PGL Z1 in a very short time period (i.e., ≤1 h for a complete infectious cycle).

### 3.6. In Vitro EP-IT22 Bacteriolytic Activity against E. amylovora

**Figure 5:**
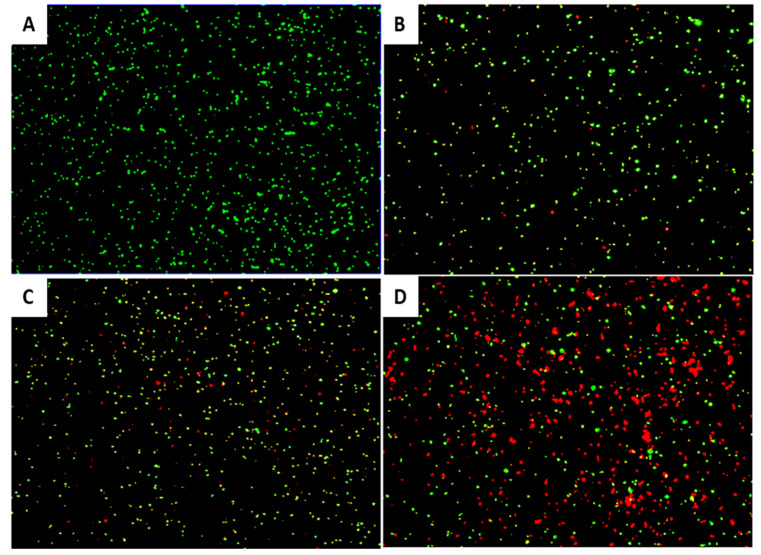
Fluorescence micrographs showing the effect of EP-IT22 on *E. amylovora* PGL Z1 cells. Green and red channels indicate live and dead cells, respectively. (**A**) untreated *E. amylovora* cells; (**B**) *E. amylovora* treated with EP-IT22 for 1 h, (**C**) 2 h, and (**D**) 8 h. Magnification 2K×.

EP-IT22 bacteriolytic activity against *E. amylovora* PGL Z1 was evaluated via FM. The results showed that after treatment with EP-IT22 (MOI = 1), fluorescence shifted from green (live bacterial cells) to red (dead bacterial cells) with increasing treatment time (Figure 5). In addition, numerous red cells were detected in the first 2 h after treatment, confirming that EP-IT22 requires only a short time to kill *E. amylovora* bacteria.

**Figure 6:**
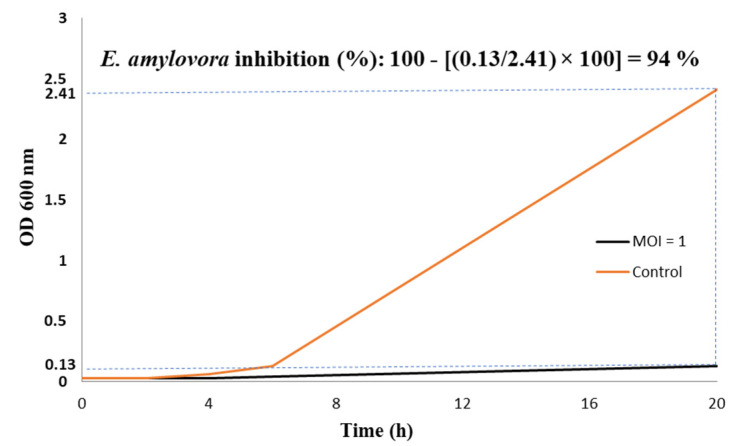
Bacteriolytic effect of phage EP-IT22 against *E. amylovora* PGL Z1 in vitro. *E. amylovora* was infected by phage EP-IT22 at a MOI of 1 and cultured for 20 h. *E. amylovora* cultures without phage were used as the control.

The turbidity assay conducted on *E. amylovora* PGL Z1 treated with EP-IT22 (MOI = 1) confirmed the significant antibacterial activity of this phage by lysing 94% of the bacterial cells (OD600 = 2.41) in 20 h (Figure 6). The slight discrepancy found between the results of live/dead imaging and those of growth kinetic values is most likely due to the nature of the assays used, whereas some of the incompletely destroyed *E. amylovora* cells can still generate a green color, although to a lesser extent compared with the fully destroyed cells.

**Figure 7:**
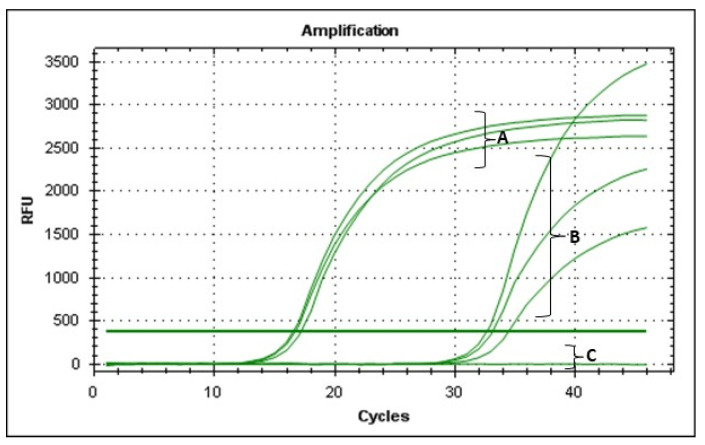
v-qPCR assay showing amplifications curves obtained from (A) untreated and (B) treated *E. amylovora* PGL Z1 cells with EP-IT22. (C) Negative control PCR reaction.

Furthermore, the V-qPCR assay agreed with the OD and FM assays, for which qPCR Ct values were >30, compared to a Ct value of 15 for untreated *E. amylovora;* this confirmed that most bacterial cells were not viable after 20 h of incubation with EP-IT22 (Figure 7). These findings strongly indicated that EP-IT22 is a potent antagonist of *E. amylovora*.

### 3.7. In Planta Biocontrol Assay

**Figure 8:**
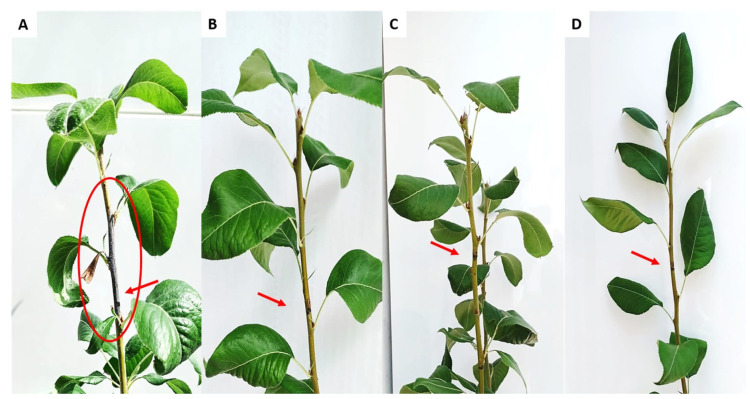
In planta assay showing the antibacterial effect of EP-IT22 on *E. amylovora* PGL Z1 infection. (**A**) *E. amylovora*-infected pear plant. (**B**) *E. amylovora*-infected plant and treated with EP-IT22. (**C**) *E. amylovora*-infected plant and treated with streptomycin sulphate. (**D**) A healthy pear plant injected with sterile water. Arrows indicate inoculation sites. The circle indicates the stem necrosis and leaf scorch symptoms.

The untreated pear plants infected with *E. amylovora* PGL Z1 showed typical symptoms of fire blight 40 days post inoculation (dpi), as the stems developed necroses and became blackened as if charred; leaves located above the inoculation sites showed wilting, scorching, and dieback symptoms (Figure 8A). On the other hand, *E. amylovora*-infected pear plants treated with EP-IT22 developed no fire blight symptoms, similarly to those treated with streptomycin sulphate (Figure 8B,C). In addition, qPCR assays conducted on leaves located above the inoculation sites showed that only the plants treated with EP-IT22 and those treated with streptomycin were negative for the presence of *E. amylovora* (Figure S2). Accordingly, the antibacterial activity of EP-IT22 was similar to that of streptomycin C in inhibiting *E. amylovora* infection in pear plants. This shows that EP-IT22 is an extremely promising antibacterial agent for the development of an ecofriendly and effective treatment for fire blight disease.

## 4. Conclusions

This study, for the first time, isolated and tested in vitro and in planta the antagonistic effect of a phage (EP-IT22) whose sequence was almost identical to the Erwinia phage Cronus reported in GenBank without any additional information on its biological characterization and potential use against fire blight. The results obtained shed light on the phage’s genome structure and features, including morphology, stability, bacteriolytic activity, and most importantly, its potential as an innovative and sustainable control agent for fire blight. Furthermore, EP-IT22 was found to be biologically safe, which offers excellent prospects for its future exploitation as a suitable and realistic antibacterial agent to include in fire blight management strategies and to replace or complement the existing agrochemicals for sustainable agriculture. Bacteriophage-based biopesticides are gaining more attention for their suitability in environmentally friendly and sustainable agriculture. They offer a promising approach to the control of plant pathogenic bacteria, which should be combined with other biocontrol methods in integrated control programs.

## Figures and Tables

**Table 1:** Bacterial strains used for determining the host range of the bacteriophage IT22.

Species	Strains	Hosts	Origins	Isolation	Lytic Activity of IT22
*Erwinia amylovora*	PGL Z1 *	*Pyrus communis*	Apulia/Italy	2013	++
*Erwinia amylovora*	PGL 102 *	*Pyrus communis*	Apulia/Italy	2013	++
*Erwinia amylovora*	OMP-BO 25.1	*Pyrus communis*	Emilia-Romagna/Italy	2000	+
*Erwinia amylovora*	OMP-BO 8630.1	*Pyrus communis*	Emilia-Romagna/Italy	2010	++
*Erwinia amylovora*	OMP-BO 1077.7	*Pyrus communis*	Emilia-Romagna/Italy	1994	++
*Erwinia amylovora*	OMP-BO 347.1	*Crataegus monogyna*	Emilia-Romagna/Italy	2006	++
*Erwinia amylovora*	OMP-BO 329.1	*Malus domestica*	Emilia-Romagna/Italy	2002	++
*Xanthomonas campestris* pv. *campestris*	CFBP 1710	*Brassica oleracea* var. *botrytis*	France	1975	−
*Pseudomonas syringae* pv. *syringae*	CFBP 311	*Pyrus communis*	Indre et Loire-France	1962	−
*Dickeya chrysanthemi*	CFBP 1346	*Chrysanthemum maximum*	Italy	1969	−

CFBP: French Collection of Phytopathogenic Bacteria, Angers, France. OMP-BO: Plant Diseases Observatory, Bologna, Italy. * Collection of CIHEAM-IAM, Bari, Italy (Valentini F., 2013; unpublished). ++: highly susceptible; +: partially susceptible; −: resistant.

## Data Availability

Not applicable.

## Supplementary Materials

The following supporting information can be downloaded at: https://www.mdpi.com/article/10.3390/v14112455/s1, Figure S1: Map of Apulia region (Italy), showing the geographical locations of sampling sites (S1-S6).; Figure S2: Host range analysis showing the bacteriolytic effect of phage EP-IT22 against *E. amylovora* strains after 20h of incubation at 25°C. *E. amylovora* strains were infected by phage EP-IT22 at MOI of 1. *E. amylovora* cultures without phage were used as the controls. Error bars represent standard deviations of three replications. Figure S3: qPCR assay performed on DNA extracted from leaves located above the inoculation sites, showing positive reactions for the presence of *E. amylovora*. (A) Untreated-*E. amylovora* infected plants 10 dpi; (B) 20 dpi; (C) 30 dpi; (D) 40 dpi; (E) genomic DNA of *E. amylovora* used as positive control; (F) EP-IT22-treated plants; (G) streptomycin-treated plants; (H) Negative control PCR reaction. Table S1: List of PCR primers used for amplifying different regions on the EP-IT22 genome. s: sense; a: antisense.
