# Supplementary material for: Identification and Characterization of Erwinia Phage IT22: A New Bacteriophage-Based Biocontrol against Erwinia amylovora
Source: Viruses. 2022 Nov 5;14(11):2455. doi: 10.3390/v14112455 (PMC9698647; doi:10.3390/v14112455)

Table S1: List of PCR primers used for amplifying different regions on the EP-IT22 genome. s: sense; a: antisense.

Primers	Sequence (5' to 3')	Position	Amplicon (bp)
EP-IT22-1	s: CACCGGTTTTCAGCACCGCC<br>a: TCACCGCATCTGGTAAGACTT	1-20<br>392-412	412
EP-IT22-2	s: GGAGCTGATAGAGGTTTTAC<br>a: TCACCGCATCTGGTAAGACTT	174025-174044<br>392-412	734
EP-IT22-3	s: CGCCTTGACAAGAGCTTCTACT<br>a: GCGAGACAGGACAATGGTTAT	12033-12054<br>12275-12295	262
EP-IT22-4	s: TCAATACCCAAGTTCCCATA<br>a: CGGTTATGCAATGTACTGTGT	15389-15408<br>15910-15930	541
EP-IT22-5	s: GGTACTCTTACCGGGAATCGA<br>a: AATCGCTAATTGATCTATTCA	66728-66748<br>67198-67218	490
EP-IT22-6	s: GCATCATTCACTTCCATTACA<br>a: CAACTAGTCGAAATCAAACCA	73445-73465<br>73900-73920	475

Figure S1. Map of Apulia region (Italy), showing the geographical locations of sampling sites (S1-S6).

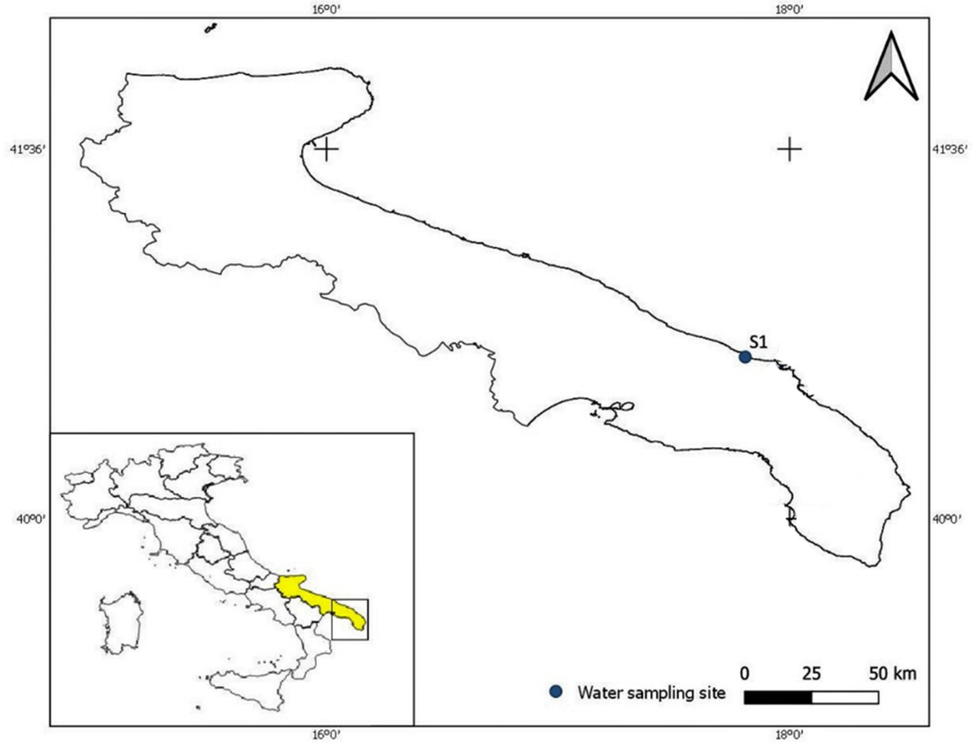

Figure S2. Host range analysis showing the bacteriolytic effect of phage EP-IT22 against *E. amylovora* strains after 20h of incubation at 25°C. *E. amylovora* strains were infected by phage EP-IT22 at MOI of 1. *E. amylovora* cultures without phage were used as the controls. Error bars represent standard deviations of three replications.

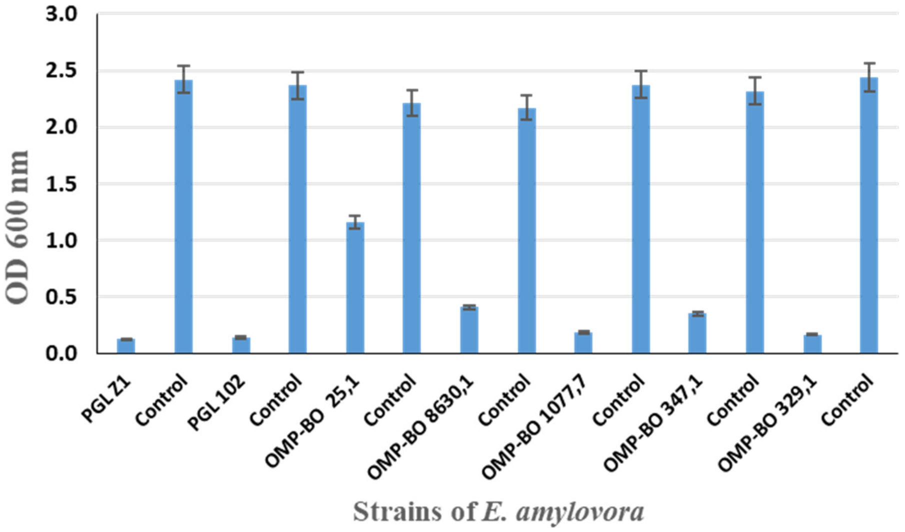

Figure S3. qPCR assay performed on DNA extracted from leaves located above the inoculation sites, showing positive reactions for the presence of *E. amylovora.* (A) Untreated-*E. amylovora* infected plants 10 dpi; (B) 20 dpi; (C) 30 dpi; (D) 40 dpi; (E) genomic DNA of *E. amylovora* used as positive control; (F) EP-IT22-treated plants; (G) streptomycin-treated plants; (H) Negative control PCR reaction.

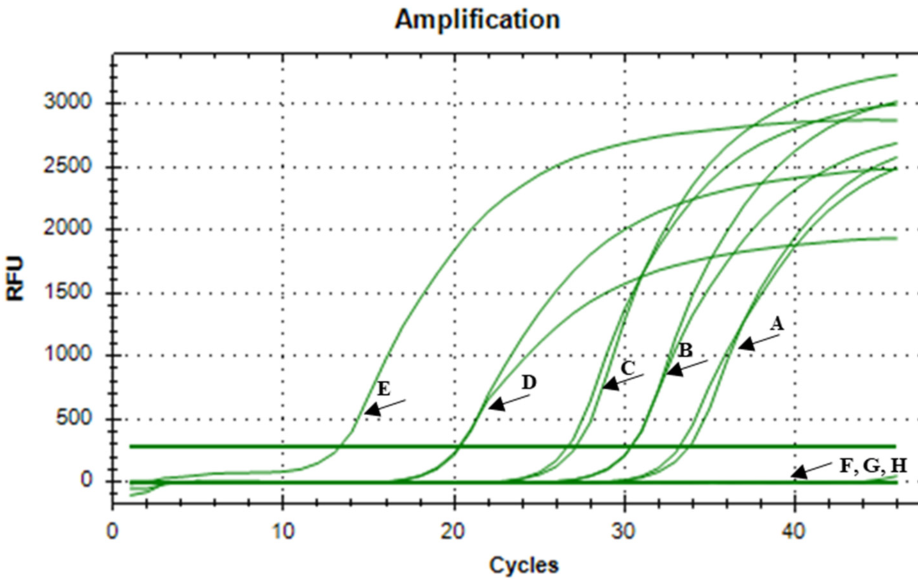

Supplement: Supplementary file 1 [file viruses-14-02455-s001.zip › viruses-1995434-supplementary.pdf]
